# Design, Control and *in Situ* Visualization of Gas Nitriding Processes

**DOI:** 10.3390/s100100218

**Published:** 2009-12-28

**Authors:** Jerzy Ratajski, Roman Olik, Tomasz Suszko, Jerzy Dobrodziej, Jerzy Michalski

**Affiliations:** 1 Institute of Mechatronics, Nanotechnology and Vacuum Technique, Koszalin University of Technology, Poland; E-Mails: roman.olik@tu.koszalin.pl (R.O.); tomasz.suszko@tu.koszalin.pl (T.S.); 2 Institute for Sustainable Technology, Radom, Poland; E-Mail: jerzy.dobrodziej@itee.radom.pl; 3 Institute of Precision Mechanics, Warsaw, Poland; E-Mail: jerzy.michalski@imp.edu.pl

**Keywords:** nitriding process, nitrided layer design, magnetic sensor, artificial intelligence

## Abstract

The article presents a complex system of design, *in situ* visualization and control of the commonly used surface treatment process: the gas nitriding process. In the computer design conception, analytical mathematical models and artificial intelligence methods were used. As a result, possibilities were obtained of the poly-optimization and poly-parametric simulations of the course of the process combined with a visualization of the value changes of the process parameters in the function of time, as well as possibilities to predict the properties of nitrided layers. For *in situ* visualization of the growth of the nitrided layer, computer procedures were developed which make use of the results of the correlations of direct and differential voltage and time runs of the process result sensor (magnetic sensor), with the proper layer growth stage. Computer procedures make it possible to combine, in the duration of the process, the registered voltage and time runs with the models of the process.

## 1. Introduction

The issues of effective design of the processes of formation of materials’ properties in the direction of multifunctional properties constitute one of the most modern world trends in the area of materials research. For this purpose, mathematical apparatus is being used more frequently and more widely and analytical and numerical mathematical models are being included together with computer intelligence methods. These tools serve to support design of surface treatment processes; among these, the gas nitriding process is of a great significance. In the recent years, it has been distinguished by a dynamically growing range of applications, chiefly in the motor and military industries, as well as in the civil aircraft industry. At the same time, requirements are increasing concerning the specific characteristics of mechanical, chemical and operational nitrided machine parts and tools. These growing requirements, in combination with an absolute condition of obtaining in a repeatable manner a complex layer structure, have served to stimulate research aimed at widening the spectrum of available process control methods [1–4]. This includes the tests covered by the present paper and concerning the development of methods to assist the process planning and development of control systems with a process result sensor added to them. The issues concerning the process result sensor have been investigated by the authors of the present paper for many years now, and the results of their analyses and tests have been already presented in numerous publications [5–11]. However, the industry demand for this manner of control, which adds a new value to the possibilities of the control of the process course has appeared only recently as a result of the abovementioned expansion of the nitriding process applications. Therefore, an interdisciplinary team represented by the authors of this article has undertaken to carry out final tests aimed at the implementation in the industry of the control system of gas nitriding process on the basis of the indications of a magnetic sensor (a process result sensor), which reacts directly in the process to the growth of the nitrided layer. An important part of the system is a designing block of an algorithm of the process parameters’ changes regarding the expected layer structure. It includes analytical mathematical and statistic models which use artificial intelligence elements to enable poly-optimization and a multi-parametrical simulation of the process course.

## 2. Structure of Nitrided Layer

**Figure 1.**
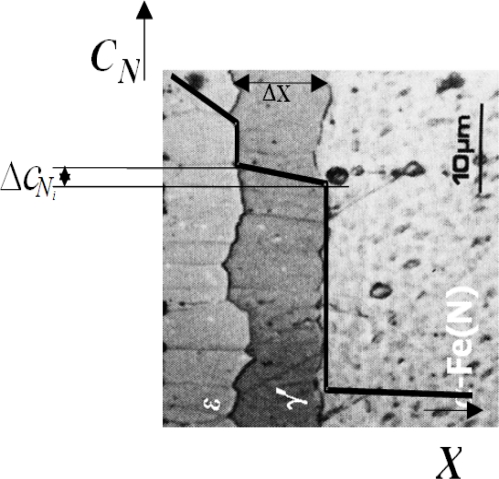
Structure of nitrided layer on iron and low-carbon steel; *C_N_*–nitrogen concentration, X–distance from surface, ε–Fe_2,3_N phase, γ′–Fe_4_N phase.

The case developed during iron gas nitriding consists of a surface zone of iron nitrides, a compound zone and a diffusion zone underneath. The compound zone which is developed at a high value of nitriding potential $K_{N}=p_{\mathrm{NH}3}/p_{H2}^{3/2}$ (the nitriding potential is directly proportional to the nitrogen activity in the gas mixture) is in accordance with Lehrer's diagram [12]: at the surface, ε phase (Fe_2,3_N) developed, while phase γ′ (Fe_4_N) lies directly adjacent to the diffusion zone (Figure 1). It is assumed that there is a local equilibrium of the nitrogen concentration at ε/γ′ interface and at γ′/diffusion zone interface [13]. Consequently, the growth kinetics of the diffusion zone is controlled by temperature only.

**Figure 2.**
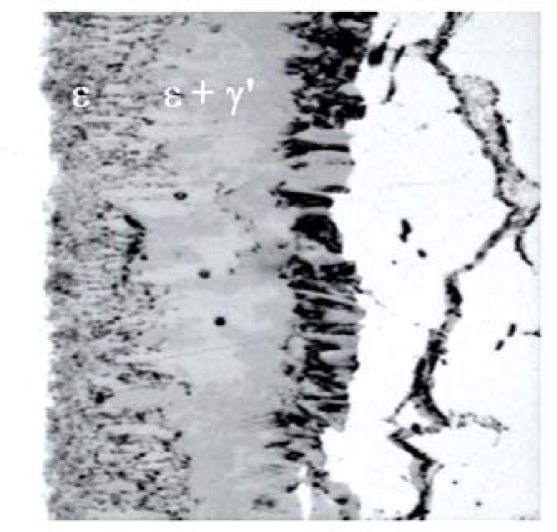
Example of the structure of layer nitrided on alloy steel.

In the case of alloy and carbon steels, the sequence of phases in the compound zone evolves with nitriding time [14,15]. The subdivisions in well-defined ε and γ′ sub-zones are replaced by a mixture of phases (Figure 2). On the basis of systematic studies regarding the phase constitution of the compound zone on carbon steels, it was concluded that a direct nucleation of ε phase on the diffusion zone was explained by a small solubility of carbon (<0.2% wt.) in γ′ phase in contrast to the large solubility for carbon in ε phase [16–21].

**Figure 3.**
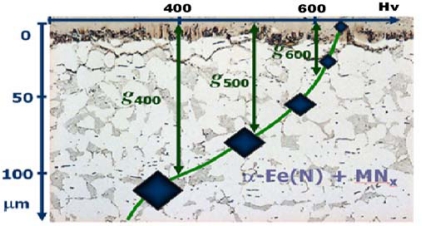
Effective thicknesses in diffusion zone.

On the basis of the research presented in papers [22,23], it was demonstrated that owing to the construction and phase composition of the iron (carbo)nitrides zone being different than in the case of iron, as well as the structural changes occurring in this zone during the process, the quasi-equilibrium of nitrogen concentration is upset on the interfacial boundary of the diffusion zone/iron (carbo)nitrides zone. Moreover, it was demonstrated that the phase structure of the iron (carbo)nitrides zone has a significant contribution, regardless of the nitrogen potential and the temperature, to the creation of the diffusion zone, and its effective thicknesses g_400_, g_500_ and g_600_ in particular (Figure 3).

The consideration presented above makes the development of the control system and development of the control system software with regard to optimal kinetics of the layer growth more challenging.

## 3. Control System

### 3.1. General Concept

**Figure 4.**
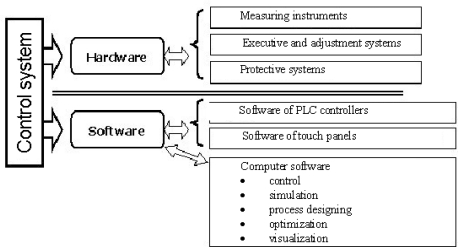
System’s general concept.

The constructed control system of the nitriding process [23] includes innovative hardware solutions and a process programming module (software) (Figure 4). The hardware, independently of the standard measuring systems of the process parameters: temperature and the composition of the nitriding atmosphere, includes the block of a process result sensor (magnetic sensor), which reacts directly in the process to the nucleation and growth of the nitrided layer. In the programming module, in compliance with the latest world trends concerning modern control systems [24–28], process models were integrated with the operating procedures. The process models which enable its simulations are of a particular importance: as a result, they provide the technologists with significant information concerning different variants of the realization of the process and the operating properties of the nitrided formed in such processes [26,27]. Consequently, operating procedures allow technologists to holistically design the nitriding process.

### 3.2. Designing Block of Process Parameters

#### 3.2.1. Guidelines

In the concept of holistic design of nitriding processes, the rules of Kansei methodology [28] were applied, and the following in particular:

- the exceptionality rule, *i.e.*, while designing the course of practically any nitriding process, it is necessary to use different values of the matrix of input parameters;
- the rule of focusing on the goals, *i.e.*, achieving the foreseen properties of the layer with the application of the smallest possible number of technological operations, e.g., changes of the temperature, the nitrogen potential or the composition of the inlet atmosphere in the duration of the process;
- the rule of the limitation of preliminary information, *i.e.*, too large a quantity of data at the start of designing results in an undesired complexity of the process;
- the rule of the use of knowledge databases, *i.e.*, for the purpose of conclusions concerning the properties of the layer, reliable data is required concerning the processes realized, especially data concerning the correlation of the values of the material’s parameters before the process and the characteristics of the nitriding atmosphere with the data concerning the operating properties of the layer after the nitriding process, collected in the database and used for the generation of knowledge databases.

The use of Kansei methodology to assist design of the nitriding process was justified by the increasing number of the required properties concerning nitrided layers in relation to machine or tool elements.

#### 3.2.2. Models of the Process

##### Analytical

Experimental data concerning the growth of particular mono-phase zones in the nitrided layer (Figure 1) [29], as well as the growth of such zones in other diffusion systems, e.g., metal-metal [30], indicate the parabolic law of the growth of phase zones:

(1) $$\Delta x_{i}=k_{i}\;\sqrt{t}$$

where:

Δ*x_i_* – thickness of i^th^ phase in n-phase layer after process time t (for the nitrided layer, the maximum value n=3),
*k_i_*– kinetic parameter of the growth of i^th^ phase, the so-called constant of the parabolic growth of i^th^ phase,
*t* – process time.

Equation (1) with experimentally designated constant *k_i_* serves to describe the growth kinetics of phase zones in a given temperature. For practical reasons, this is enough in most cases, as the knowledge of *k_i_* value in different temperatures for all the phases of the diffusing system makes it possible, in accordance with equation (1), to determine the change of the thickness of a given Δ*x_i_* phase in the process time function. However, this is an oversimplified description of the growth kinetics, which makes it impossible to determine, in the case of a nitrided layer, e.g., nitrogen diffusion coefficients in individual layer phases or nitrogen concentrations at phase borders. One cannot foresee on the basis of this equation the influence of the remaining phases creating the layer upon the speed of the growth of a given phase, either. For this reason, it became necessary to develop general mathematical equations describing dependencies between growth parameters (k_i_) and diffusion parameters, which facilitated the determination of diffusion coefficients in mono-phase layer zones, as well as forecasting of the phase growth in the function of the process time. In the model developed, whose particulars were given in paper [31], the final result is the equations given below (2), which in a direct manner connect the kinetic parameter of a given phase k_i_ with the difference of concentrations on the phase borders and an effective diffusion coefficient in the phase:

(2) $$\sum_{j=1}^{n}\;\alpha_{i,j}\;k_{i}\;k_{j}={\left(D_{i}^{c}\right)}_{\mathrm{ef}}\;\Delta c_{i}\;\;i=1,2,3$$

where:

$$\alpha_{i,j}=\left\{\begin{array}{l} {\bar{c}}_{i},\;i>j\\ \frac{1}{4}\left(3{\bar{c}}_{i}+c_{i,i+1}\right),\;i=j\\ {\bar{c}}_{j},\;i<j \end{array}\right\}$$

*k_i_*–kinetic parameters of the growth of i^th^ and j^th^ phase,
Δ*c_i_*–difference of concentrations of the diffusing element on the border of i^th^ phase.

This is a system of non-linear equations, which can be solved with e.g., the method of successive approximations (iterations):

(3) $$k_{i}=\frac{2{\left(D_{i}^{c}\right)}_{\mathrm{ef}}\;\Delta c_{i}}{\sum_{j=1}^{n}\;\alpha_{\mathrm{ij}}\;k_{j}}\;\;i=1,2,3\;\;1-\varepsilon ,\;\;2-\gamma^{\prime },\;3-\alpha$$

In the method of successive approximations, e.g., for zone *ε*, the following dependencies are obtained:

(4) $$k_{\varepsilon }^{\left(1\right)}=\frac{2{\left(D_{\varepsilon }^{c}\right)}_{\mathrm{ef}}\;\Delta c_{\varepsilon }}{\sum_{j=1}^{3}\;\alpha_{\mathrm{ij}}\;k_{j}^{\left(0\right)}},\;\;k_{\varepsilon }^{\left(2\right)}=\frac{2{\left(D_{\varepsilon }^{c}\right)}_{\mathrm{ef}}\;\Delta c_{\varepsilon }}{\sum_{j=1}^{3}\alpha_{\mathrm{ij}}\;k_{j}^{\left(1\right)}}$$

From the system of equations obtained, it is evident first of all that the growth parameters are greater the bigger difference of concentration at the borders of phases Δc_i_ is, in accordance with the diagram of the phase equilibrium, as well as the greater the effective diffusion coefficient is in a given phase– ${\left(D_{i}^{c}\right)}_{\mathrm{ef}}$. This model can also be applied for the calculations of the thickness growth of the whole zone of (carbo)nitrides on steels, in particular on those stages of the process when phase ε dominates.

##### Statistical models based on artificial intelligence methods

Due to the previously mentioned (items 1 and 2) phenomena and mechanisms which form the growth of the nitrided layer, it is difficult to construct software for the control systems of a process which guarantees that a layer with the required and repeatable operating properties is obtained. Therefore, there is an increasing interest in the use of artificial intelligence methods in computer assisting of the designing of layer constitution technologies.

The article covers a section of the research concerning the application of evolutionary algorithms for designing of the gas nitriding process parameters, which guarantees obtaining the required hardness profile (proper effective thicknesses) of the nitrided layer. Evolutionary algorithms are especially recommended for solving difficult optimization tasks. Therefore, these mathematical procedures are frequently used to solve numerous problems in materials engineering which occur in a wide range of such issues as the following:

- modelling of materials,
- simulations of nanocrystals and atomic/molecular clusters,
- optimization of metallurgical processes,
- designing/search for new materials with the required properties,
- supervision of the growth processes of single coats and multilayer coatings,
- an analysis of the physical and structural properties of thin-layer systems.

For the purpose of the creation of a model of the process, the results were used of experimental research conducted in the past several dozens of years at the Koszalin University of Technology, the Institute for Sustainable Technology in Radom and the Institute of Precision Mechanics in Warsaw.

**Figure 5.**
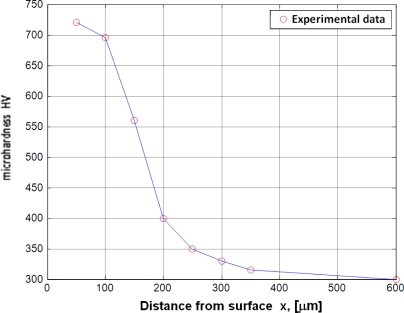
Example of microhardness profile for 18H2N2 (18CrNi8) steel (T_2_ = 530 °C (803 K), t_2_ = 480 min, K_N_ = 6).

They specify the impact of the main parameters of the process (temperature T, time t and nitrogen potential K_N_) and of the chemical composition of steel (the atomic concentration of alloying elements forming nitrides) on the hardness profiles (cf. Figure 5, Table 1).

**Figure 6.**
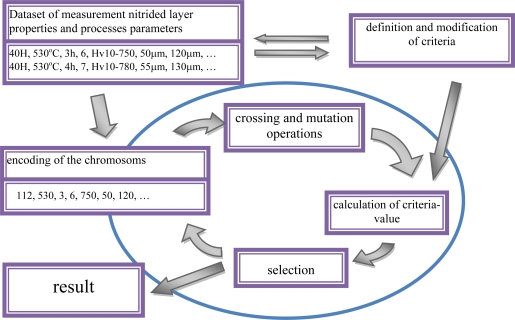
Idea of the procedure of evolutionary algorithms.

Owing to the data sets collected in the database, a statistical model could be developed, where the procedure of evolutionary algorithms was used (Figure 6). This model made it possible to forecast the process parameters for the required hardness profile in the diffusion zone. In the model under development, each chromosome includes encoded parameters of a single steel nitriding process. Chromosome sets form the so-called population. For each chromosome, a steel hardness profile is designed corresponding to it. For this purpose, a trained neural network is used, which performs the role of a model. Each chromosome (a set of parameters) is then evaluated, which consists in the determination of the matching of the corresponding hardness profile to the profile sought. On the further step, the chromosomes undergo a selection (a selection of chromosomes with the best matching), and a modification with the aid of crossing and mutation operations. Then they are promoted to another population. In this manner, by way of a directed evolution of steel nitriding parameters, such a set of chromosomes is selected for which the hardness profile matches best the profile sought.

In the model, the correctness of the solution sampled is determined on the basis of the error which is specified with a suitably trained artificial neural network (ANN). The mutation process was realized in such a way that those individuals which are better adapted (*i.e.*, those for which the error calculated on the basis of the ANN model is the smallest) have the greatest impact on it.

A reverse error propagation type network was used for the construction of the neural model. This is one of the most popular neural networks where the number of the possible values of neurons in the output layer falls within the range (0; 1.0) or (−1.0; 1.0). It is true that this network requires the use of the uncomfortable data encoding procedure, but as consequence its training process is shortened by many times.

The differentiation in the values of hardnesses resulted in the need to divide the data into several groups. The tests demonstrated that the most accurate representations of profiles were obtained by dividing the data according to the base (steel) type. This also led to a substantial reduction of the time (to several minutes) required to train the neural network to any base type. It should be noted, however, that for some grades of steel, the data sets collected which relate to the process parameters with their results, are still too little for the indications of the neural network (once it has been trained) to be reliable. Additionally, due to the small space of the database, training of ANN in some cases occurs without the determination of the test part. Therefore, the network is trained on the basis of the whole data set, without checking the correctness of the “deduction” for the testing set which has not been separated.

In order to minimize the oscillations and fluctuations in the attempts to obtain a global minimum of the error function, the network training algorithm, *i.e.*, the reverse error propagation algorithm, was parameterised with the aid of the learning coefficient and the network moment coefficient. For this purpose, an adaptation mechanism of these coefficients was used which was developed by Chan and Falside [32]. It was dynamically applied in each learning iteration.

**Figure 7.**
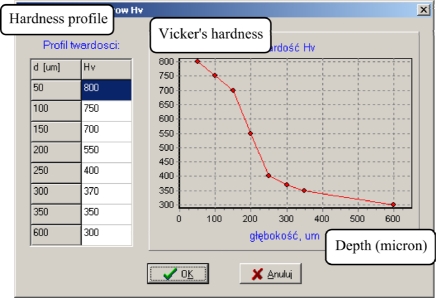
Dialogue window for entering input data (hardness profiles) for an evolutionary algorithm of seeking optimal process parameters.

**Figure 8.**
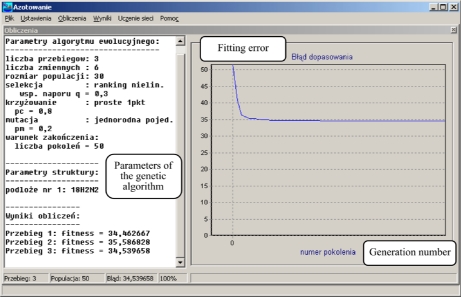
Window of the programme for the search of the variation area of the process parameters. The diagram presents the dependence of the error matching of the generation number of the set of parameters.

**Figure 9.**
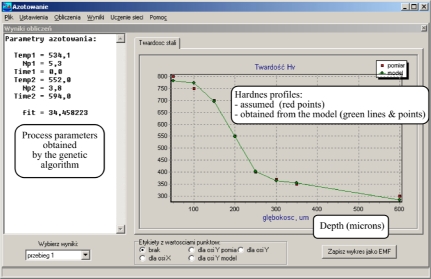
A window presenting the final result of the operation of the evolutionary algorithm: a set of process parameters which give as a result the profile which matches best the required profile.

The tool developed makes it possible to determine the parameters of a process which guarantees obtaining the required hardness profile in the nitrided layer. The steel grade and the required profile hardness in the nitrided layer are given on the input of the programme. We obtain the values of the main process parameters (Figures 7–9) on the output. As a model for the nitriding process is used in neural network trained and based on the collected experimental data.

### 3.3. Visualization and Control of the Process

**Figure 10.**
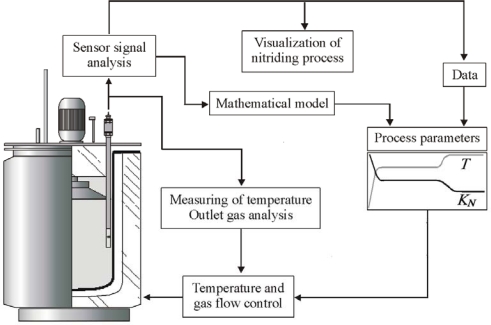
Schematic diagram of the automatic system of the nitriding process with the visualization system for the course of the layer growth.

As regards the nitriding process control systems used at present, attempts are made to obtain increasingly precise methods to determine the composition of the nitriding atmosphere. The sensors used in the latest solutions of automatic systems make it possible to determine the most representative quantity which has an impact on the course of the creation of the layer, *i.e.*, the nitriding potential (K_N_) whose algorithm of changes in the function of the process time and temperature constitutes the point of reference for the control system. It is determined on the basis of the indications of a thermo-conductometric sensor of hydrogen content, or the indications of an ammonia content sensor, whose working principle is based on absorption spectroscopy. However, a selection of a suitable algorithm of the changes of the nitrogen potential and the temperature as well as an application of increasingly better sensors of the nitriding atmosphere composition and temperature does not always guarantee that the expected result is achieved. For various practical reasons, the process may not run as expected. In such cases, we learn about a layer structure being contrary to the one expected only after the completion of the process. Hence, in the light of the growing demands for nitrided elements, concerning above all an increase of their life, or growing demands concerning a precise repeatability of the process results, it is indispensable to construct a system which works on the basis of the indications of the process result sensor. This sensor makes it possible either to directly monitor the process course and a correction of the changes of the process parameters in the case of its improper course, or to control the process on the basis of the indications of the layer growth sensor. Therefore, it becomes justifiable to add a block which represents directly in the process a growth of the nitrided layer (Figure 10) to the process control and the adjustment system.

#### 3.3.1. Selection of the Measuring Method

While a nitrided layer is being formed, both electric properties of the nitrided material: resistivity (*ρ*) and magnetic properties change. As regards the magnetic properties, relative magnetic permeability (*μ*_r_) and the intensity of magnetic coercive field (H_c_) are most sensitive to phase and structural changes [33–36].

One of the chief parameters which decide the value of the resistivity is the mean free path of conduction electrons. During formation of the diffusion zone, it gradually decreases as a result of the growth of the concentration of dispersion media: interstitial nitrogen atoms and the forming nitrides of alloying elements.

In the case of magnetic properties, the diffusion zone is ferromagnetic, while the surface zone of iron nitrides (compound zone) is paramagnetic. In the ferromagnetic diffusion zone, magnetic permeability and the intensity of the magnetic coercive field are the function of above all the state of macro- and microstresses in this layer. It is accepted that macrostresses are the result of the nitrogen concentration gradient; the nitrides of alloy elements are responsible for the development of micro-stresses.

In the nitriding process, the area covered by the above-mentioned changes of the electro-magnetic properties constitutes a small fraction of the thickness of the nitrided materials. This is the main factor that influenced the choice of investigating these changes by the induction method. The material specimens tested by this method are placed within an alternating electromagnetic field created by a coil. This induces eddy currents that in turn create their own field, directed opposite to coil field, thus limiting its depth of penetration into the bulk of the material.

**Figure 11.**
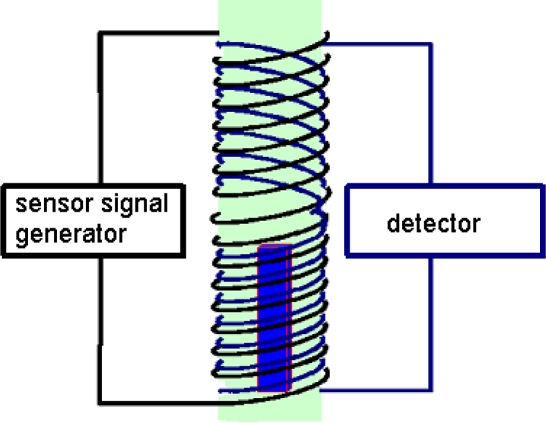
Schematic diagram of two-winding sensor.

In the solution applied, the measuring section of the sensor was equipped with a coil with double winding. A high frequency alternate current flowing through the primary winding generates an electromagnetic field which penetrates the sample tested. As a consequence, the resultant field is formed in the coil area, which induces a voltage signal in the secondary winding of the coil. As a result of the testing examinations, the variant was selected with two push-pull connected sections of the secondary coil, while the sample tested is located in one of the sections: cf. Figure 11. Owing to this, a change of the voltage signal induced depends solely of the changing electromagnetic properties of the sample.

Finally, the voltage induced in the measuring winding of the coil is the function of the following parameters [35]:

(5) $$\hat{U}={\hat{U}}_{0}\left(1-\eta +\eta \mu_{r}{\hat{\mu }}_{\mathrm{sk}}\right)$$

where:

*Û*_0_– voltage induced in the measuring winding when the coil is empty,
η– coil filling factor,
*μ*_r_ – relative magnetic permeability,
*μ̂_sk_* – effective permeability: its introduction makes the value of the magnetic field intensity independent from the distance from the surface of the sample.

The value of the voltage induced in the measuring winding depends directly on the relative magnetic permeability (*μ*_r_) and indirectly on the resistivity, which is a parameter of effective permeability (*μ̂_sk_*). Moreover, by decreasing the specimen thickness, it is possible to reduce the extent of the changes in the induced voltage values owing to the resistivity [35].

Therefore, in order to monitor the growth of the nitrided layer with the use of a magnetic sensor, and so in order to assign the changes of the voltage induced in the coil measuring winding to the suitable stage of the layer creation, one needs to know the following representation: 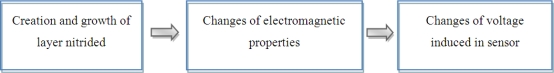


On the first stage of the formation of the nitrided layer, diffusion of nitrogen causes an expansion of the surface layer of the material. This expansion is being counteracted by the remaining part of the material with a smaller nitrogen concentration. Macrostress profiles develop as a result of these actions; for those specimens that, from the point of view of diffusion, are of finite thickness, these stresses can be described by the equation [37]:

(6) $$\sigma \left(x,\;t\right)=\frac{\beta \cdot E}{1-\nu }\{\left[\bar{N}(t)\right]-\left[N(x,t)\right]\}$$

where:

σ(x,t) – macrostresses which are parallel to the surface, in x distance from the surface,
β - Vegard constant,
E - Young's modulus
ν - Poisson constant,
[N̄ (t)] - average nitrogen concentration in sample in t time of process,
[N(x,t)] - surface nitrogen concentration in x distance from surface in t time of process.

Owing to the fact that the expansion of the lattice induced by diffusing nitrogen is positive (β > 0), it follows from equation (6) that near the surface, there are compressive stresses {[*N̄* (t)] < [*N* (x,t)]}, while tensile stresses exist in the areas which are further away from the surface where {[*N̄* (t) ] > [N(x,t)]}. The abovementioned formula, when the changes of the nitrogen concentration profiles in the process time function are known, makes simulative generation of macrostresses possible. Therefore, in compliance with relation (5), the changing values of the voltage induced in the sensor can be compared from the qualitative perspective with the change of the profiles of macro-stresses in the process time function, with a close connection between the magnetic permeability and the stresses being assumed. At the same time, nitrogen concentration profiles can be obtained by solving the phenomenological Fick's law of diffusion, assuming that the interfacial chemical reaction is dominant in the initial period of the formation of layers. This implies that the surface concentration of nitrogen achieves an equilibrium value with the nitriding atmosphere relatively slowly.

**Figure 12.**
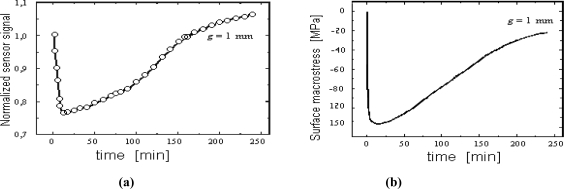
(a) Variation in sensor signal (magnetising current frequency f = 150 kHz) as a function of nitriding time for an iron specimen of thickness g = 1 mm. (b) Calculated changes of surface compressive macrostress in an iron specimen of thickness g = 1 mm; process parameters: T = 560 °C (833 K), K_N_ = 0.08.

Figure 12a presents an example course of the changes of the voltage induced in the magnetic sensor registered in the nitriding process of a sample made from Armco iron and with 1 mm thickness, in an atmosphere with a nitrogen potential (K_N_ = 0.08) located in the area of phase α on Lehrer's diagram [T = 560 °C (833 K)]. The registered characteristics U = f (t) correspond in a qualitative sense to the changes of surface macro-stresses (Figure 12b) as calculated for the above-mentioned parameters of the process. These macro-stresses represent two characteristic stages of the creation of the diffusion zone. The former is connected with an increase of the surface nitrogen concentration accompanied by an increase of compressive stresses and a decrease of the voltage signal, while in case of the latter stage there is a relaxation of stresses, which causes an increase of the voltage.

#### 3.3.2. Design, Testing and Implementation of the Measuring Block

The most important element in the designing of a generator was the selection of such a frequency which conditions a specific penetration depth of the electromagnetic field into the sample. The correct penetration depth of the field guarantees the uniqueness of the development of stresses in the diffusion zone of the nitrided layer as well as an optimal sensitivity to the nucleation and the growth of the thickness of the compound zone (iron nitrides zone). However, to make the induced voltage signal reflect the changes in surface macrostresses accurately, the area $S_{\sigma }\left(S_{\sigma }=\int_{0}^{d}\;\sigma_{s}\;\mathrm{dx},\;\;\sigma_{s}=\sigma (0,t)\right)$ contained under the stress profile and situated in the area of electromagnetic field penetration (d – the electromagnetic field penetration depth) should be achieving its maximum value at the time as the maximum values are obtained for the stress (σ_s_):

(7) $$\text{max}\;S_{\sigma }\Leftrightarrow \text{max}\;\sigma_{s}$$

This condition having been met, in the process time corresponding with the obtaining of the maximum value by surface macrostresses (σ_s_), the minimum of the voltage induced in the sensor should be observed.

**Figure 13.**
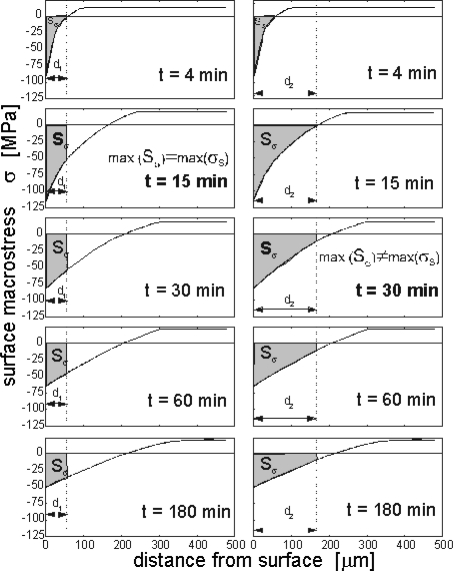
Examples of calculated profiles of macrostresses in the diffusion zone for given process times: electromagnetic field penetration depth d_1_ corresponds with frequency f_1_, d_2_ – frequency f_2_ (f_2_ < f_1_).

Figure 13 presents examples of the calculated profiles of macrostresses σ_s_ for different times of the process with such parameters which condition the formation of diffusion zone αFe(N) only. In this figure, surface S_σ_ is marked under the profile of stresses for two electromagnetic field penetration depths. The presented results of calculations indicate that condition (7) is fulfilled for higher frequency f_1_ (f_1_ > f_2_), for which the field penetration depth is d_1_ (d_1_ < d_2_). Any frequencies higher than f_1_ will also fulfill relation (7), but in this case the sensitivity will be reduced of the reaction of the voltage system due to the decrease of value *μ̂_sk_*. For any frequencies lower than f_1_, condition (7) will not be fulfilled.

**Figure 14.**
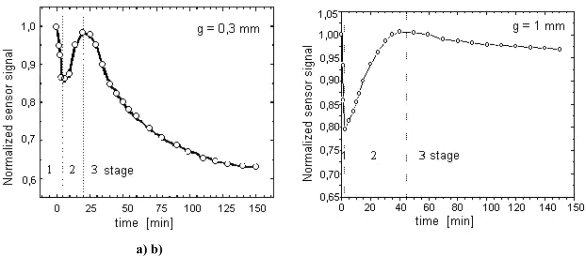
Variation in sensor signal as a function of nitriding time for iron specimens with given thicknesses: process parameters T = 560 °C (833 K), K_N_ = 0.9.

The interpretation presented above of voltage-time characteristics concerned those processes where only a ferromagnetic diffusion zone was formed. When the nitrogen potential of the atmosphere (K_N_) exceeds the threshold value for the formation of phase γ′ or ε, a paramagnetic surface zone (compound zone) is formed in the process temperature which consists of the abovementioned iron nitrides. The increase in the thickness of this zone, with an appropriate selection of specimen thickness and magnetising current frequency (see below), will cause a drop in the signal value. It is evident on the basis of an analysis of the development of surface macrostresses in the initial stage of layer formation that the time to obtain the maximum value for this stress is equal to the time for the surface nitrogen concentration to reach maximum solubility in αFe lattice, for a given temperature. If the time needed to obtain the maximum surface concentration is taken to be equivalent to the γ′ nitride incubation time, there should be a strict correlation between the time needed to achieve maximum values for surface compressive stress and γ′ phase incubation time, and the time necessary to obtain a minimum voltage induced in the sensor. Therefore, in these processes, a relaxation of stresses in the diffusion zone will be accompanied by the surface being covered with γ′ or ε + γ′ nitride phases. Providing these nitrides do not form a compact zone, their impact on the recorded voltage signal will be insignificant with regard to the increase in the signal induced by the relaxation of stresses in the diffusion zone. It is extremely important, however, from the point of view of nitride zone (compound zone) control, for this effect to be easily distinguishable as the moment in the process when a compact zone of these nitrides is formed. This condition can be fulfilled by a suitable selection of the thicknesses of the samples placed in a magnetic sensor and the frequency of magnetising current, which conditions a specific penetration depth of the electromagnetic field in the material. One cannot achieve a measurable impact of the compound zone on the induced signal solely by an optimization of frequencies for any thickness of the samples. This is so as the zone due to its magnetic properties (μ_r_ ≅ 1) becomes, as compared with a ferromagnetic diffusion zone (μ_r_ ≫ 1), practically transparent for the electromagnetic field. As a result of this, together with an increase of the compound zone thickness, the field moves into the material. This issue is illustrated in Figure 14 on the example of voltage-time courses registered for various frequencies and thicknesses of the samples.

These graphs were obtained for samples made from Armco iron with the thicknesses of 0.3 mm and 1 mm, nitrided in an atmosphere with potential K_N_ = 0.9 (a nitrogen potential is located in the area of phase γ′ on Lehrer’s diagram) and temperature 560 °C (833 K). Two magnetizing current frequencies were used: 50 kHz for a sample 0.3 mm thick and 150 kHz for a sample 1 mm thick. As it can be seen in Figure 14a, now a third stage occurred in the presented changes of the induced voltage for the sample 0.3 mm thick. This stage reflects the growth of the compound zone. For a sample with this thickness and the accepted frequency (f = 50 kHz) in the process time, in which the voltage signal induced reaches a maximum value (the start of the 3^rd^ stage), the thickness of the compound zone is 3–4 μm. For a 1 mm thick sample (Figure 14b), even making the frequency three times as high (150 kHz) did not increase the sensitivity of the voltage signal to the increase of the paramagnetic nitrides zone (compound zone). This means that in order to explicitly represent in the registered signal the growth of the compound zone, from the start of the creation of a compact zone with a minimal thickness, it is necessary to optimize at the same time the magnetising current frequency and the thicknesses of the samples.

#### 3.3.3. Design and Construction of Magnetic Sensor

**Figure 15.**
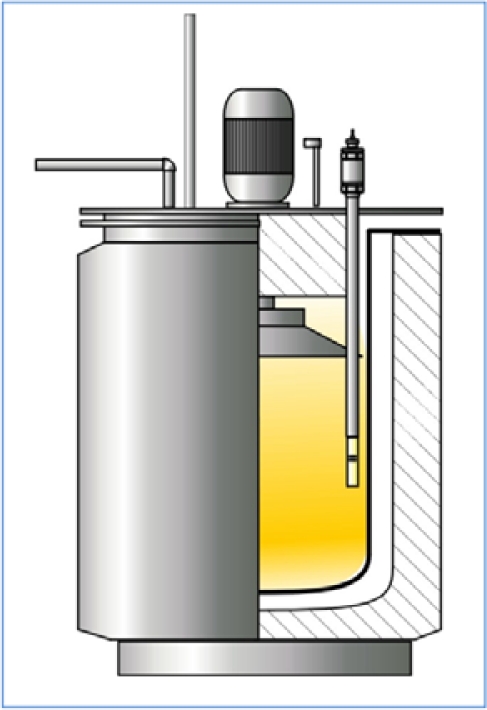
Magnetic sensor placed in furnace retort.

The basic part of the measuring system, *i.e.*, the magnetic sensor, is placed in a furnace retort, cf. Figure 15. This is a run-through sensor: the gas nitriding atmosphere flows through it and causes the formation of a nitride layer on the specimen placed inside the sensor.

#### 3.3.4. Development of Computer Models for *in Situ* Visualizations of the Layer Growth Kinetics

By introducing necessary simplifications concerning phenomenological models which describe physical phenomena which occur in the gas nitriding process, but in compliance with the rules of an eidetic analysis, the following key research issues were solved:

- dependencies were identified between the process parameters and the layer structure for an *in situ* visualization of the layer growth kinetics and for the control of the process which ensures an optimal layer due to its expected physical and chemical properties, and in particular its surface hardness as well as the thickness and the morphological composition;
- complementary cooperation was developed between the mathematical model and the indications of the magnetic sensor which registers *in situ* the formation of the nitrided layer.

**Figure 17.**
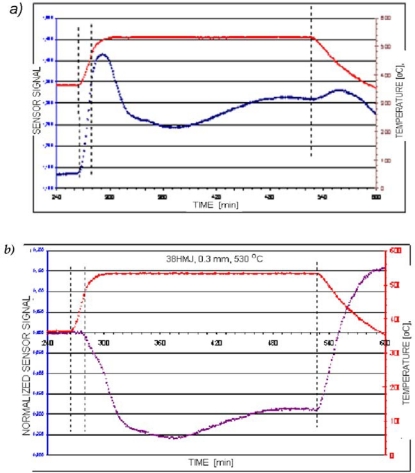
Variation in sensor signal as a function of nitriding time for steel 38HMJ (41CrAlMo7) specimens. (a) sensor signal without background temperature compensation, (b) sensor signal with background temperature compensation.

Particular attention was paid to the development of a model allowing the separation of the background temperature. It is the impact of changes in electromagnetic properties of steel samples on the sensor signal, caused by variations in temperature that occurs during the heating furnace to the temperature of the process. The background temperature is characteristic of each individual steel and must be subtracted from the sensor signal by the corresponding software of the system. It is developed based on a database containing the sensor signal values recorded during the heating of samples to the process temperature. Samples of these processes are protected from the nitriding process. A sample variation in the sensor signal with background temperature, and with no background is presented in Figures 17a,b. After subtracting of the background, during the heating furnace until the start of the formation of a layer, which initiates the beginning of the dissociation of ammonia [ca. 350 °C (623 K)], the signal recorded by the sensor does not change.

**Figure 18.**
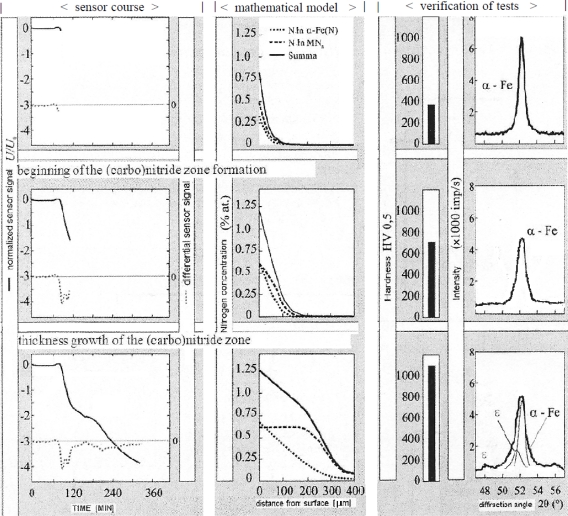
Presentation of the functioning of the control system which consists in a complementary cooperation of the magnetic sensor signal and the mathematical model. The nitriding process of 38HMJ (41CrAlMo7) steel; process parameters T = 560 °C (833 K), K_N_ = 2.1.

In particular, such models were developed which make the following possible (Figure 18):

- an isolation of the background temperature from the signal registered with the magnetic sensor,
- an identification of the nucleation moment of nitrides,
- a detection of the further stages of the layer creation,
- a quantitative determination of the layer growth kinetics.

An application of computer models developed to enable obtaining quantitative information about the current characteristics of the layers, *i.e.*, the thickness of the compound zone, surface hardness and hardness profiles are shown on the example of steel 38HMJ (41CrAlMo7) (Figure 18), because products made from the steel often undergo a process of nitriding.

## 4. Discussion and Conclusions

The present paper presented two complementary approaches to the construction of an intelligent control system for the gas nitriding process. The first one consists in developing mathematical models of the process and modern databases as well as expert systems to support the operator in making decisions concerning the selection of specific changes in the parameters of the process. The other one additionally uses specially constructed sensors, which placed directly in the process react to the growth of the layers and their structures.

Computer aiding of designing of the surface layer constituting technologies (analytical and numerical mathematical models, artificial intelligence methods) with required and repeatable operational properties constitutes one of the key issues of surface layer engineering. Attempts are made to forecast the properties of surface layers on the basis of the parameters of the base material and the process environment characteristics. Forecasting procedures enable one to analyze different variants of processes and to programme a technology which is optimal considering the criteria accepted concerning the surface layer properties. An implementation of such a concept for a specific surface finishing makes it possible to replace a very costly trial and error method, which is used at present in practice. However, this requires a solution of numerous complex scientific and application problems concerning modeling of confounded functional dependencies between the surface layer properties and the process environment characteristics, and the base material.

From among a wide range of the methods developed to assist the development of software for the block to control the nitriding process, the paper presents an analytical model which represents the growth kinetics of the mono-phase zones of the nitrided layer on iron and low carbon steels, as well as a model based on evolutionary algorithms which make it possible to select the parameters of a process which guarantees the required hardness profile in the nitrided layer. Independently of this, a sequence was presented of actions aimed at an effective use of the changes of the magnetic properties of the nitrided material, so that the layer growth kinetics could be represented during the process. The purpose of these two procedures is to obtain an intelligent process control system, from the phase of the construction of software for the operation system to the control of the process on the basis of the result sensor (a magnetic sensor).

The unique value of the operation systems is constituted by the possibility to monitor the growth of the nitrided layer on all its development stages as a result of a complementary combination of the indications of the sensor with mathematical models.

## Figures and Tables

**Figure 16.**
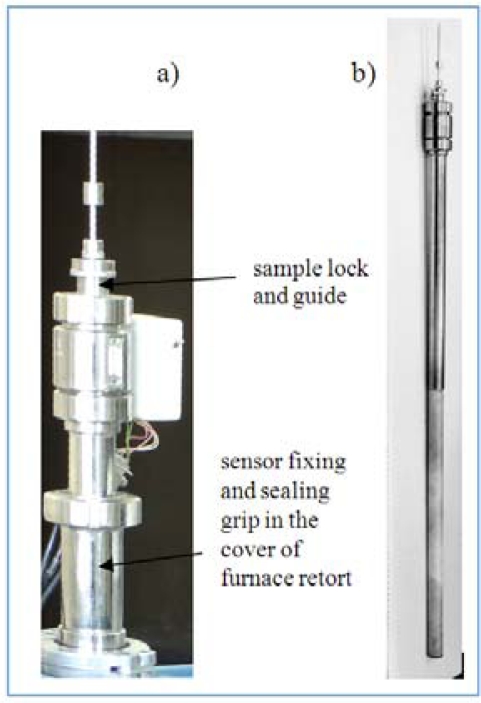
Photos which present the magnetic sensor: (a) sensor head with a grip, (b) view of the sensor.

**Table 1.** Characteristics of experimental data used in modelling.

	Steel			Number of samples	Range of parameters							
	EN	W.NR	PN		First degree				Second degree			
					q-ty	T_1_[^°C^]	t_1_ [min]	K_N_	q-ty	T _2_[^°C^]	t_2_ [min]	K_N_
1	18CrNi8	1.5920	18H2N2	10	-	-	-	-	10	530–570	120–780	3.2–6
2	0.2%C,1%Mn,1.2%Cr,0.1%Ti,0.3%Si		18HGT	85	20	480–580	120–270	1.15–30	85	480–580	25–3090	0.4–30
3	20MnCr5	1.7147	20HG	23	-	-	-	-	23	530–590	120–1920	2.75–4.5
4	25CrMo4	1.7218	25H3M	10	4	530	240–270	5.25–11.5	10	530	120–3090	0.6–11.5
5	∼25CrMo4	∼1.7218	25H5M	2	1	530	270	5.25–7	2	530	780–3090	0.8–5.25
6	30CrMoV9	1.7707	4340	32	-	-	-	-	32	530–590	120–1920	2.75–4.5
7	33CrMoV12-9	1.8519	33H3MF	35	1	530–570	270	1.5–30	35	530–570	120–3090	0.8–30
8	35CrAlMo5	1.8506	35CrAlMo5	3	-	-	-	-	3	530–570	480	2.75–4.4
9	0.4%C,1%Mn,1.2%Cr,0.3%Ni,1.2%Si		35HGSA	2	2	540	360	20	2	540–550	3240	0.7–0.9
10	34CrAl6	1.8504	34CrAl6	22	-	-	-	-	22	530–570	120–1920	3–4.5
11	∼35CrMo8	∼1.2312	36H3M	6	-	-	-	-	6	550	720	1.5–30
12	38CrMoV21	∼1.2343	∼WCL	32	-	-	-	-	32	530–590	120–1920	2.75–4.5
13	41CrAlMo7	1.8507	38HMJ	44	19	480–580	120–480	1.1–30	44	480–580	25–3240	0.4–30
14	41Cr4	1.7035	40H	43	3	500–570	60–270	1.5–30	43	500–570	120–3090	0.8–30
15	42CrMoV73	1.7741	40H2MF	2	1	530	270	5.25–7	2	530	780–3090	0.8–5.25
16	∼42CrMo4	∼1.7225	40HM	4	-	-	-	-	4	560–570	240–540	0.85–3.1
17	42CrMo4	1.7225	4140	15	2	530–570	270–450	0.85–7	15	530–570	120–3090	0.8–6.1
18	30CrMoV9	1.7707	4340	30	14	480–570	120–270	3.2–20	30	480–570	25–3090	0.8–20
19	C45	1.0503	45	50	1	500–590	270	3–7	50	500–590	120–3090	0.8–4.75
20	X40CrMoV511	1.2344	WCLV (H13)	1	-	-	-	-	1	500	360	5.1
21	1%C,0.7%Cr,0.7%Mn,0.6%Ni, 0.2%Si		IMPACTO	8	-	-	-	-	8	530–570	120–720	3.2–6.1
22	1.7%C,12%Cr,0.3%Mn,0.3%Ni		NC10	3	-	-	-	-	3	530–570	480	2.75–4.4
23	42CrMo4	1.7225	4140 (SPS)	8	-	-	-	-	8	530–570	120–720	3.2–6.1
24	∼HS6-5-2	∼1.3343	SW3S2	5	5	530	240	9.25	5	530	120–1920	0.6
25	X37CrMoV51	1.2343	WCL	16	6	530–570	240–270	1.5–30	16	530–570	120–3090	0.6–30
		TOTAL		491	79	480–590	60–480	0.85–30	491	480–590	120–3090	0.4–30
